# Outcomes after (chemo)radiotherapy for anal cancer – A nationwide cohort study

**DOI:** 10.1016/j.ctro.2026.101163

**Published:** 2026-04-06

**Authors:** C.L. Deijen, M. Berbée, E.D. Geijsen, K.J. Neelis, L. Wee, M.A.B. Bakker-Van der Jagt, A.H. Boer, H.M. Ceha, J.S. Cnossen, M.P.W. Intven, M.M. Leseman-Hoogenboom, G. Mulder-Ebrahimi, K. Muller, V. Oppedijk, O. Reerink, H. Rütten, P.H. Spruit, B. van Triest, On behalf of the LPRGE Dutch Platform for Radiotherapy in Gastro-Enterology

**Affiliations:** a Department of Radiation Oncology, Amsterdam University Medical Centres, location VUmc, Amsterdam, the Netherlands; b Department of Radiation Oncology, Netherlands Cancer Institute, Amsterdam, the Netherlands; c Department of Radiation Oncology (Maastro), GROW Research Institute for Oncology and Reproduction, Maastricht University Medical Centre+, Maastricht, the Netherlands; d Department of Radiation Oncology, Amsterdam University Medical Centres, location AMC, Amsterdam, the Netherlands; e Department of Radiation Oncology, Leiden University Medical Centre, Leiden, the Netherlands; f Department of Radiation Oncology, University Medical Centre Groningen, Groningen, the Netherlands; g Department of Radiation Oncology, Haaglanden Medical Centre, The Hague, the Netherlands; h Department of Radiation Oncology, Catharina Hospital, Eindhoven, the Netherlands; i Department of Radiation Oncology, University Medical Centre Utrecht, Utrecht, the Netherlands; j Zuidwest Radiotherapeutisch Instituut, Roosendaal, the Netherlands; k Instituut Verbeeten, Tilburg, the Netherlands; l Radiotherapiegroep, Deventer, the Netherlands; m Radiotherapy Institute Friesland, Leeuwarden, the Netherlands; n Department of Radiation Oncology, Isala Clinic, Zwolle, the Netherlands; o Department of Radiation Oncology, Radboud University Medical Centre, Nijmegen, the Netherlands; p Department of Radiation Oncology, Northwest Clinics, Alkmaar, the Netherlands

**Keywords:** Anal cancer, Squamous cell carcinoma, Treatment, Outcomes, Chemotherapy, Radiotherapy, Chemoradiotherapy, Risk factors

## Abstract

**Introduction:**

Due to the rarity of anal cancer, real-world data on treatment outcomes are scarce. However, its incidence is increasing in the Netherlands with > 300 new cases and 70 deaths yearly. Standard of care consists of chemoradiotherapy (CRT) or radiotherapy alone (RT). The primary objective of our nationwide study was two-fold; to evaluate real-world outcomes after curatively intended CRT/RT for anal squamous cell carcinoma (ASCC), and to create a large, high-quality dataset as a national benchmark to guide future prospective research.

**Material and methods:**

Data were collected retrospectively from 16 Dutch institutions treating ASCC between 2015 and 2018. Primary endpoint was locoregional recurrence free survival (LRFS). Secondary endpoints were overall survival (OS), disease specific survival (DSS), colostomy free survival (CFS), complete response (CR) and toxicity.

**Results:**

A total of 462 patients were analysed. Most patients received CRT (85%), with mitomycin-capecitabine as a radiosensitizer in 86% of cases. After median follow-up of 5 years, LRFS for all patients was 80% at 3 years and 78% at 5 years. In total 82% of patients had reached CR, with a median interval to define CR of 3 months. At 3 years, OS, DSS and CFS were 78%, 86% and 88%, respectively.

**Conclusion:**

These real-world data demonstrate that curatively intended CRT/RT for ASCC results in LRFS of 80% at 3 years. Organ preservation outcomes were favourable with a CFS of 88% at 3 years. These findings support the current treatment approach as the reference standard for these patients and provide a national benchmark to guide future prospective research.

## Highlights

• (C)RT for anal cancer results in LRFS of 80% and CFS of 88% at 3 years.
• estimated median overall survival (OS) time was 6.9 years.
• negative risk factors for LRFS and CFS were: greater tumour size and HPV negative tumour status; for CFS additional WHO ≥ 2.
• negative risk factors for OS were: WHO ≥ 2, tumour stage IIIb-IV and HPV negative tumour status.
• there was no institutional case load effect for LRFS, OS or CFS.

## 1 Introduction

Anal cancer is a rare type of cancer according to the definition of the EUROCAN network.[1] However, the incidence in The Netherlands is current increasing, with more than 300 new patients in 2024 and approximately 70 deaths.[2] In the majority of cases, histology indicates squamous cell carcinoma (SCC); other less frequently occurring types include adenocarcinoma, small cell carcinoma and melanoma. In 80–85% the anal carcinoma is associated with human papillomavirus (HPV) infection, specifically types 16 and 18. Other risk factors include human immunodeficiency virus (HIV), use of immunosuppressive drugs and smoking.[3], [4], [5], [6], [7] In addition, history of cervical, vulvar, or vaginal cancer are also related to an increased risk of anal cancer.[8].

In 30–40% of the patients, lymph node metastases are found at primary diagnosis. Distant metastases at first presentation are observed less frequently, in 5–8% of patients. For patients presenting with stage I disease, the 5-year overall survival is > 90%, in case of stage IV disease, the survival rate decreases to 18%-36% at 5 years.[2], [9].

Until approximately 50 years ago, treatment of anal cancer consisted of extensive surgery (abdominal perineal resection), with significant morbidity. Radiotherapy (RT) showed inferior outcomes and was not incorporated in standard of care.[10] During following decennia the chemotherapy regimen with mitomycin-C (MMC) and 5-fluoro-uracil (5-FU) was introduced as neoadjuvant treatment. Because of the unexpected high rate of complete response found after resection and preserving organ and function became an important aspect of treatment, a new treatment modality was formed: definitive chemoradiotherapy (CRT).[11] In the years following, this standard organ preserving therapy was further improved by replacing 5-FU with oral capecitabine and new RT techniques (development of Intensity Modulated Radiotherapy (IMRT) and Volumetric-Modulated Arc Therapy (VMAT), minimising dose on the surrounding healthy tissue and toxicity).[12], [13], [14] The past years the role of immunotherapy is being studied, especially in advanced disease. Also the combination of RT and immunotherapy is subject of ongoing trails.[15], [16], [17].

Preferably, treatment is discussed in a multidisciplinary team, including a radiation oncologist, medical oncologist, surgical oncologist, radiologist and pathologist. Only in tumours ≤ 2 cm located in the *peri*-anal margin without lymph node or distant metastases, treatment can be limited to a wide local excision of the tumour. For larger tumours, or in case of locoregional lymph node metastases, standard of care consists of CRT with MMC and 5-FU (or capecitabine) or RT alone.[4], [10], [12], [18].

As mentioned above, an important issue in treatment of anal carcinoma is preserving the function of the anus, without compromising local tumour control. However, local control varies widely between studies and depends on certain risk factors, such as HPV and p16 positivity in the tumour.[10], [19].

In conclusion, anal cancer is a rare disease and nationwide results of treatment are scarce. The primary objective of our retrospective chart study was two-fold: first, to evaluate real-world outcomes after curatively-intended CRT/RT for anal SCC (ASCC) and evaluate prognostic factors on these outcomes; second, to create a large high-quality dataset as a national benchmark to guide future prospective research.

## 2 Patients and methods

### 2.1 Patients

Data were retrieved retrospectively − via treatment chart audit by local institutional principal investigators (PIs) − from all patients treated with curative intent in 16 different Dutch institutions between January 2015 and January 2018 (i.e. start date of treatment must be in 2015, 2016 or 2017). Inclusion criteria for statistical analysis were: Patients with either biopsy proven anal carcinoma or clinical suspicion of anal carcinoma − based on clinical examination and/or diagnostic imaging and/or high grade dysplasia in biopsy − and treated with (C)RT with curative intent (including definitive treatment with (C)RT of local anal cancer recurrences or residual disease after surgery).

The primary endpoint of this study was locoregional recurrence free survival (LRFS) at 3 years, defined as no clinical or radiological evidence of cancer recurrence in the pelvic or perineal area (calculated from last day of treatment to last follow-up date or date of local recurrence).

Secondary endpoints were:

− LRFS at 5 years (calculated as time from last day of treatment to last follow-up date or date of local recurrence).
− Disease-specific survival (DSS) at 3 and 5 years (calculated as time from last day of treatment to last follow-up date, date of death or date of recurrence).
− Distant metastasis free survival (DMFS) at 3 and 5 years (calculated as time from last day of treatment to last follow-up date, date of death or date of metastasis).
− Overall survival (OS) at 3 and 5 years (calculated as time from last day of treatment to last follow-up or date of death).
− Colostomy-free survival (CFS) at 3 and 5 years (calculated as time from last day of treatment to last follow-up date, date of death or date of colostomy).
− Complete response (CR) at 3 and 5 years (calculated as time from last day of treatment to determination complete response).
− Exploratory predictive analyses on age, sex, performance status (WHO), T stage, N stage, disease stage (regarding AJCC TNM7), RT dose, chemotherapy given, HPV, p16, HIV, SCC antigen and case volume effect.
− Toxicity grade ≥ 3 according to a predefined list and the CTCAE v4 or 5 (according to local protocols).

### 2.2 Ethical review

Primary approval was obtained from the Internal Review Board at the Netherlands Cancer Institute in Amsterdam, the Netherlands (IRBd20-330). Additionally, all participating centers obtained local ethical approval for retrospective data collection from their own institutions.

### 2.3 Data collection

A centralised private data repository was set up by the lead PI institution, Netherlands Cancer Institute and data were collected in the collection system ALEA. The required data fields were set up as electronic case report forms, and PIs from participating institutions were given login-password authenticated access to their own forms. PIs were briefed at a kickoff meeting, and consensus was shared about how to extract data values from their respective treatment charts. The local PIs were responsible for populating the eCRFs. The eCRFs were compiled and exported as a set of plaintext files by the data manager at ALEA.

### 2.4 Statistical analysis

R version 4.5.1 was used for statistical analysis, with p < 0.05 (one tailed) considered to be significant. For LRFS, DFS and OS Kaplan-Meier estimates were used. Predictive factors were analysed with chi square tests, T-tests, Pearson or Spearman correlations and Cox regression.

### 2.5 RT dose and target volumes

In The Netherlands, in the primary setting a RT dose of 59.4–64.8 Gy on the gross tumour volume (GTV) and 45 Gy on the clinical target volume (CTV) in 33–36 fractions is prescribed (using a surdosage or a simultaneous integrated boost). RT is combined with MMC and 5-FU or capecitabine on RT days in all tumours, except for T1N0 with ≤ 1 cm transmural invasion. In case of postoperative RT, different schedules are applied, ranging from 54-59.4 Gy on the GTV and 36–45 Gy on the CTV in 25–33 fractions. At the moment, the national guideline is under revision, amongst others with the aim to reach consensus for RT schedules postoperatively.

The GTV consists of all visible and palpable tumour, based on imaging and physical examination. The CTV consists of GTV plus margin (5–10 mm), plus the elective pelvine lymph nodes (including inguinal, iliacal internal/external, obturator, mesorectal and presacral lymph nodes). In specific cases the CTV is adjusted (e.g. when tumour is only located in the rectum, excluding inguinal lymph nodes or in case of small tumours not invading the rectum, excluding presacral lymph nodes).

## 3 Results

### 3.1 Patient and tumour characteristics

There were 505 individual patients in terms of the number of sealed eCRFs at time of database lock. A total of 462 patients that matched the aforementioned inclusion criteria were analysed in detail; the summary characteristics are provided in (Table 1). The vast majority of included patients were treated in institutions with medium (average 25–39 patients per inclusion period) and high (average ≥ 40 patients per inclusion period) caseloads. Patient numbers were relatively stable from year to year, averaging just over 150 patients per annum in the period 2015–2017, inclusive. There was a marginally higher incidence in women (53%) relative to men (47%). Median follow-up time was 5 years. The median age was 63.5 years. In most of the patients biopsy showed squamous cell carcinoma (97%), in a minority only high grade dysplasia (1%). The 9 other histologies included were: basaloid squamous cell carcinoma (6), undifferentiated carcinoma with squamous and neuroendocrine components (1), atypical papillomatous hyper- and parakeratotic squamous cell lesion (1), undifferentiated large cell carcinoma probably squamous cell differentiation (1). These were recorded as free text comments in the database; however, these patients were treated as de facto ASCC, thus they were included in the statistical analyses.

**Table 1:** Patient and tumour characteristics.

	**N (%)**
**Total number of patients analysed**	462
**Institutional case load**	
Low (0–24 patients per inclusion period)	78/462 (17)
Medium (25–39 patients per inclusion period)	199/462 (43)
High (≥40 patients per inclusion period)	182/462 (39)
*1 centre with incomplete patient entry*	3/462 (1)
**Year of starting treatment**	
**2015**	149/460 (32)
**2016**	158/460 (34)
**2017**	153/460 (33)
**Not recorded**	2
**Age at start treatment (median)**	63.5 years (range 27–90)
**<50 years**	51/460 (11)
**50**–**70 years**	285/460 (62)
**>70 years**	124/460 (27)
**Not recorded**	2
**Sex**	
**Female**	246/462 (53)
**Male**	216/462 (47)
**WHO Performance Status**	
**0**–**1**	412/454 (91)
**2**	37/454 (8)
**3**	5/454 (1)
**Not recorded**	8
**Histological subtype**	
**High-grade dysplasia**	6/462 (1)
**Squamous cell carcinoma**	447/462 (97)
**Not one of the above**	9/462 (2)
**T stage**	
**T1**	57/462 (12)
**T2**	248/462 (54)
**T3**	107/462 (23)
**T4**	50/462 (11)
**N stage**	
**N0**	247/457 (54)
**N1**	84/457 (18)
**N2**	75/457 (16)
**N3**	51/457 (11)
**Nx**	5
**M stage**	
**M0**	457/461 (99)
**M1**	4/461 (1)
**Mx**	1
**AJCC Staging Version 7**	
**I**	46/457 (10)
**II**	185/457 (40)
**IIIa**	82/457 (18)
**IIIb**	140/457 (31)
**IV**	4/457 (1)
**Unknown**	5
**HIV status**	
**Positive**	26/190 (14)
**Negative**	164/190 (86)
**Not recorded**	272
**HPV or p16 status**	
**Either HPV or p16 or both positive**	124/157 (79)
**Either HPV or p16 or both negative**	33/157 (21)
**HPV positive and p16 negative**	2/157 (1)
**HPV negative and p16 positive**	3/157 (2)
**Not recorded**	305
**SCC (µg/L)**	
**≥1.5**	20/38 (53)
**<1.5**	18/38 (47)
**Not recorded**	424
**Primary tumour maximum length (median)**	40 mm (range 1–90)
**Tumour location**	
**Anal canal**	284/462 (61)
**Peri-anal skin**	44/462 (10)
**Both (anal canal and*peri*-anal skin)**	134/462 (29)

The majority of tumours were staged T2 (54%), N0 (54%), and M0 (99%). HPV and/or p16 status was reported in 157 patients, of which 124 patients were classified positive (79%). In 190 patients HIV status was reported, of which 26 were HIV positive (14%). The median tumour length was 40 mm, the majority of which were located either in the anal canal only, or in the anal canal with extension to the *peri*-anal skin.

### 3.2 Diagnostics

Regarding diagnostic work-up, in most of the patients MRI-scan, digital rectal exam, CT-scan and PET-scan were performed (Table 2). In 211 patients inguinal ultrasound was performed, of which in 153 cases a cytological biopsy was done. Of these 153 biopsies, 63 (41%) were tumour positive and 89 (59%) were negative.

**Table 2:** Diagnostics and treatment details.

	N (%)
**Diagnostic work-up**	
MRI-scan	435/462 (94)
Digital rectal exam	429/462 (93)
**CT-scan**	290/462 (63)
**PET-scan**	285/462 (62)
**Colono-/sigmoido-/proctoscopy**	250/462 (54)
Inguinal ultrasound	211/462 (46)
Chest X-ray	22/462 (5)
Other	37/462 (8)
**Cytological biopsy inguinal lymph nodes**	153
Positive	63/152 (41)
Negative	89/152 (59)
Not recorded	1
**Chemotherapy**	
**No chemotherapy**	67/462 (15)
Not indicated	41/67 (61)
Indicated	26/67 (39)
Chemotherapy*	395/462 (85)
*MMC only*	
*Capecitabine only*	2/393 (0.5)
***5FU only***	7/393 (2)
***MMC + capecitabine***	3/393 (0.5)
***MMC + 5-FU***	338/393 (86)
*MMC + capecitabine + 5-FU*	41/393 (10)
*Not recorded*	2/393 (0.5)
	2
Chemotherapy per stage*	
I	4/46 (9)
II	169/185 (91)
IIIA	79/82 (96)
IIIB	134/140 (96)
IV	4/4 (100)
**Radiotherapy dose**	
Primary tumour (median)	59.4 Gy (range 30.0–70.0)
Pathological lymph nodes (median)	59.4 Gy (range 30.0–66.0)
Elective lymph nodes (median)	49.5 Gy (range 23.4–56.0)
**Radiotherapy technique**	
3D conformal	4/462 (1)
Electrons	8/462 (2)
Intensity modulation (IMRT)	171/462 (37)
Volume modulated arc (VMAT)	268/462 (58)
IMRT/VMAT plus brachytherapy boost	11/462 (2)

*i.e. concurrent chemoradiotherapy.

### 3.3 Treatment

The majority of the patients received CRT (85%), with MMC-capecitabine as a radiosensitizer in 86% of these patients (Table 2*)*. The median RT dose given to the primary tumour was 59.4 Gy (range 30.0–70.0) and to pathological lymph nodes 59.4 Gy as well (range 30.0–66.0). The median elective RT dose was 49.5 Gy (range 23.4–56.0) (supplementary Fig. S1).

Of patients with stage I disease, 9% received concurrent chemotherapy (CRT) and 91% received RT alone. Of patients with stage II disease 91% received CRT, of patients with stage IIIA 96% and stage IIIB 96%. All 4 patients with stage IV received concurrent chemotherapy (CRT) *(*Table 2*)*. Overall, in 67 patients no chemotherapy was given at all. In 41 patients with a small tumour there was no indication for chemotherapy following national guidelines (i.e. T1 tumours ≤ 1 cm transmural invasion and N0. In all other cases, standard of treatment is CRT). In 26/67 patients there was an indication for chemotherapy. Reasons that these patients underwent RT only were poor performance status, age and comorbidity.

Regarding RT schedules used postoperatively or in case of an irradical resection (N = 44), there was wide variety in dose given (supplementary Fig. S2).

### 3.4 Outcomes

#### 3.4.1 LRFS, DSS, DMFS, OS

**Fig. 1:**
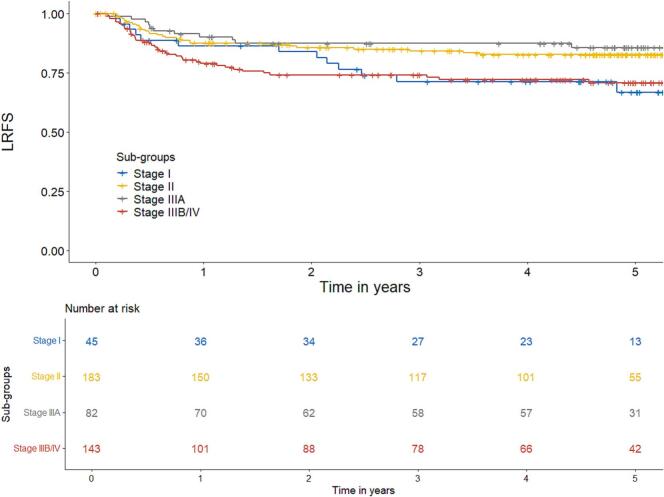

LRFS for all patients was 80% at 3 years and 78% at 5 years (Fig. 1). When analysed per stage, LRFS for stage I, II, IIIA and IIIB/IV was 67%, 83%, 86% and 71%, respectively at 5 years.

**Fig. 2:**
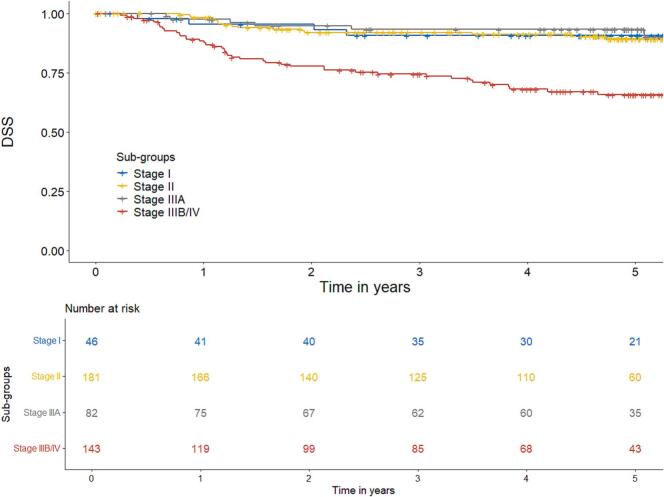

DSS was 86% at 3 years and 82% at 5 years. For stage I, II, IIIA and IIIB/IV DSS was 91%, 89%, 93% and 66%, respectively at 5 years (Fig. 2).

At 3 and 5 years the DMFS rates were 89% and 86%, respectively. For stage I, II, IIIA and IIIB/IV these rates were 88%, 93%, 93% and 71% at 5 years, respectively.

**Fig. 3:**
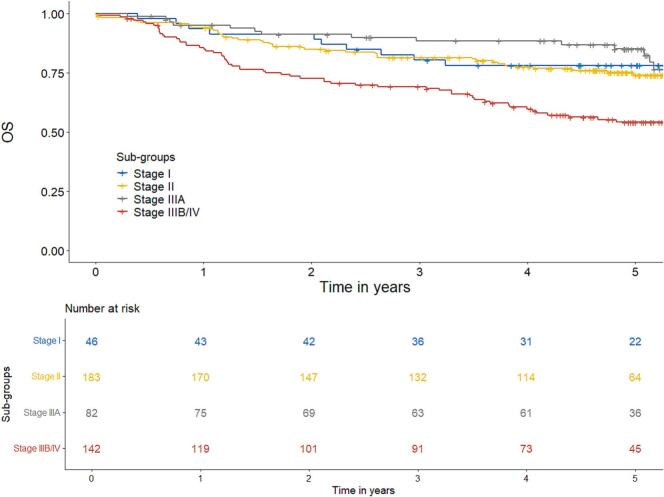

OS rate was 78% at 3 years and 70% at 5 years. When analysed per stage, OS for stage I, II, IIIA and IIIB/IV was 78%, 74%, 85% and 54%, respectively at 5 years (Fig. 3). In total 151 patients died during follow-up. Of these, 74 patients died as result of ASCC.

#### 3.4.2 CFS, CR

**Fig. 4:**
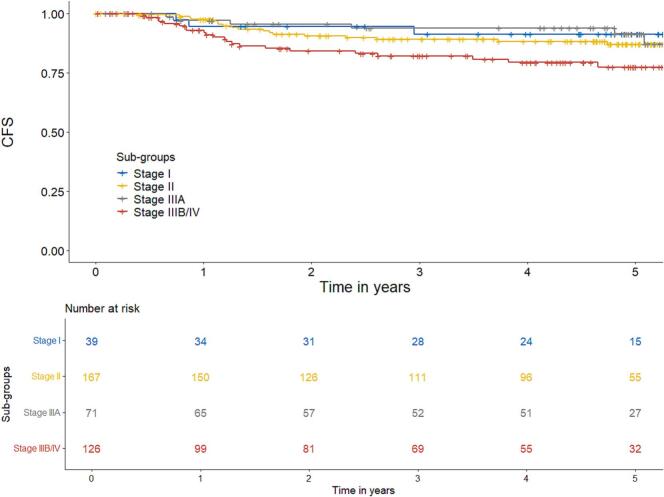

At 3 years the CFS was 88% and at 5 years 85%. At 5 years rates for stage I, II, IIIA and IIIB/IV were 91%, 87%, 91% and 77%, respectively (Fig. 4).

In total 374 out of 460 patients (82%; data from 2 patients were missing) reached CR at some point in time, with a median interval to registering CR of 3 months after treatment. Digital rectal examination was the most commonly-used CR assessment method (N = 343), followed by MRI (N = 168) and PET (N = 124).

The estimated median OS time was 6.9 years, median had not yet been reached for LFRS, DSS, DMFS or CFS.

#### 3.4.3 Prognostic factors LRFS, OS and CFS

In univariate analyses, prognostic factors for LRFS were: male, T3-4, stage IIIb-IV, no chemotherapy, greater tumour size, higher dose on elective nodes and HPV negative tumour. For OS prognostic factors were higher age, male, WHO ≥ 2, T3-4, N+, stage IIIb-IV, no chemotherapy, greater tumour size, higher dose on elective nodes and HPV negative tumour. Prognostic factors for CFS in univariate analyses were medium case load, WHO ≥ 2, T3-4, stage IIIb-IV, greater tumour size and HPV negative tumour. Regarding CR < 6 months, in univariate analyses prognostic factors were male, high case load, T3-4, stage IIIb-IV, greater tumour size, higher dose on elective nodes and HPV negative tumour.

In multivariate analyses greater tumour size (HR 1.43) and HPV negative tumour status (HR 4.22) remained risk factors for LRFS and WHO ≥ 2 (HR 3.32), stage IIIb-IV (HR 3.49) and HPV negative tumour status (HR 3.09) for OS. For CFS WHO ≥ 2 (HR 6.60), greater tumour size (HR 1.37) and HPV negative tumour status (HR 11.07) remained risk factors in multivariate analyses. For CR < 6 months there were no prognostic factors in multivariate analyses (Supplementary Table S1-S4).

#### 3.4.4 Toxicity

The most frequent reported acute side effect (i.e. during and < 3 months after treatment) grade ≥ 3 was dermatitis, which was reported 357 times (*peri*-anal 242; inguinal 115). Grade ≥ 3 diarrhea was reported in 33 patients and proctitis in 30 patients (supplementary Table S5).

Regarding late side effects (i.e. > 3 months after treatment), most frequent grade ≥ 3 reported were proctitis (40 patients), pain (39 patients), insufficiency fracture (17 patients) and fecal incontinence (17 patients) (supplementary Table S6). Dyspareunia in female and erectile dysfunction in male were scored as yes or no, and were reported 30 and 17 times, respectively.

## 4 Discussion

This study represents one of the largest national cohorts reporting real data on outcomes in patients treated with curatively intended CRT/RT for ASCC. With participation of 16 institutes, the majority of institutes in The Netherlands treating patients with anal cancer with RT contributed. Data from IKNL, the Dutch nationwide cancer registry, showed in the period 2015–2017 685 patients diagnosed with stage I-III anal cancer. Hence, we expect that approximately 74% (505/685) of all eligible Dutch patients were included in our cohort.

In a minority of patients included in our study only high grade dysplasia was found at biopsy. In these patients the clinical course, in combination with imaging and high grade dysplasia was considered suspicious enough to treat these patients for anal carcinoma. We did not explicitly record in the database whether the patients in whom only high grade dysplasia was found had been treated for high grade dysplasia previously. However, from our clinical experience in The Netherlands, patients are not treated with (C)RT for only having high grade dysplasia (recurrence) without other concurrent factors indicating anal carcinoma.

As one of the main goals of treatment of ASCC is anal preservation, we chose LRFS as primary endpoint. At 3 years LRFS was 80%, resulting in a high CFS rate of 88%, which is comparable to rates described in literature.[20], [21], [22], [23] We tried to find risk factors for local failure and as described in literature, HPV is a strong predictor for treatment response. HPV negative tumours are in general more resistant to RT, resulting in poorer outcomes after CRT.[13] In our study HPV negative tumour status indeed was a risk factor for both the surrogate endpoints for poor local response LRFS and CFS, but not for CR in multivariate analyses. Unfortunately, in The Netherlands not in all patients HPV status is determined, as this has no impact on the choice of treatment and HPV status was therefore only known in 157 patients. Regarding LRFS, also greater tumour size was associated with worse outcome. For CFS, negatively associated factors were HPV negative tumour status, WHO ≥ 2 and greater tumour size. The OS rate of 70% at 5 years is in line with other studies.[20], [21], [22], [23], [24] We found several factors resulting in worse outcome: WHO ≥ 2, stage IIIb-IV and HPV negative tumour.

Remarkably, outcomes for stage IIIA (T1-3 N1 or T4N0) were better than for the other stages. This finding was also apparent for DFS by another single centre retrospective study conducted in The Netherlands.[20] We hypothesised that these observations could be due to differences in use of chemotherapy together with RT for the different stages. However, the rate of patients with stage IIIA disease who received chemotherapy (96%) was comparable with stage II (91%) and stage IIIB (96%). Although patient numbers are small, further improvements in treatment outcome for other stages than IIIA seems warranted.

To improve oncological results, as well as functional outcomes, the PLATO study group (PersonaLising Anal cancer radioTherapy dOse) initiated several trials studying RT dose escalation and de-escalation. ACT3 focusses on risk-adapted use of adjuvant low-dose CRT in anal margin tumours, in ACT4 reduced-dose CRT in early anal carcinoma is evaluated and ACT5 is a dose-escalation study with CRT in locally advanced ASCC [25].

Recently the study group presented their long-term data for the PLATO-ACT4.[19] In this study patients with early-stage anal cancer (T1–2 (≤ 4 cm) N0–NxM0) were randomised to reduced dose (41.4 Gy in 23 fractions) and standard dose CRT (50.4 Gy in 28 fractions). At 3 years, the locoregional failure rate was 16.4% in the standard treatment arm and 12.4% in the experimental group. In our study locoregional failure was 20% at 3 years, which is comparable to the standard treatment arm in the PLATO-ACT4 study.

Three-year CFS was 85.5% and 93.2% and OS 92.6% and 98.1% in the standard and experimental arms, respectively.[26] CFS in our study at 3 years was again comparable with 88%. However, we found an OS rate of 78% at 3 years, which is substantially lower than the OS results in the PLATO-ACT4 study. A difference between our study and the PLATO-ACT4 is that the median RT dose given to the primary tumour in our study was 59.4 Gy. Patients in the PLATO-ACT4 received 50.4 Gy in the standard arm and 41.4 Gy in the experimental arm and patients in the standard arm had worse OS. These differences in OS may suggest a potential negative impact of higher radiation dose, although confounding factors cannot be excluded. The observed differences in OS may be explained by differences between the cohorts, such as the higher number of patients with a WHO performance score of 2 or higher and an higher median age in our cohort, and other differences between trial and real world data. Furthermore, it is reported in literature that CRT can cause lymphocytopenia, which can result in poorer outcomes, amongst others worse DFS and OS.[27], [28], [29] Other potential mechanisms of action may include increased risk of high grade toxicity, resulting in treatment breaks or complete discontinuation of treatment for example. Finally, it can be speculated that in case of salvage surgery, the risk of post-operative complications may be higher after high dose RT.

Treatment of anal cancer is available in almost all RT institutes in the Netherlands and performed by a dedicated team. Delineation guidelines and planning-instructions have been reviewed and updated recently in a national team project to ensure the quality of treatment nationwide. Whether the interesting results of the PLATO-ACT4 should be changing practice in the Netherlands is currently subject of debate.

Another subject of debate is the role of immunotherapy in the curative setting. Several studies are evaluating the role of immunotherapy combined with CRT. One study (RADIANCE) is investigating treatment with durvalumab and CRT in locally advanced HPV positive ASCC. Another trial (CORINTH) is evaluating safety and tolerability of pembrolizumab and CRT. Finally, the ongoing ECOG-ACRIN 2165 is treating patients with nivolumab after CRT in high-risk stage II-IIIB ASCC [15], [16], [17].

This study has several limitations. Data were collected retrospectively and some variables, e.g. determination of CR and reporting of toxicity, were probably subject to interpretation of the physician. Moreover, not every side effect is always scored if not addressed during follow-up. Furthermore, risk factors as HPV and HIV were reported in less than half of the patients and SCC level in less than 10 percent of the patients. Given these small numbers it is difficult to perform robust analyses. Finally, these study was initiated in 2020. Because of the necessity to achieve 5-year follow-up, inclusion period was limited until 2018 with the intention to report outcomes as soon as possible. However, collecting and completing all data from 16 different centres was a lengthy process, resulting in a delay of reporting the outcomes.

In conclusion, real-world data from a large Dutch population study demonstrate that curatively-intended CRT/RT for ASCC results in LRFS and CR rates of 80% and 82% at 3 years, respectively. Organ preservation outcomes were favourable with a CFS of 88% at 3 years. These findings provide a robust national benchmark for the treatment of anal SCC and may act as a reference standard for future studies aiming to individualise treatment based on biological and clinical risk factors.

## CRediT authorship contribution statement

**C.L. Deijen:** Writing – review & editing, Writing – original draft, Project administration, Investigation, Data curation, Conceptualization. **M. Berbée:** Writing – review & editing, Writing – original draft, Investigation, Data curation, Conceptualization. **E.D. Geijsen:** Writing – review & editing, Writing – original draft, Investigation, Data curation, Conceptualization. **K.J. Neelis:** Writing – review & editing, Writing – original draft, Investigation, Data curation, Conceptualization. **L. Wee:** Writing – review & editing, Writing – original draft, Visualization, Investigation, Formal analysis, Data curation, Conceptualization. **M.A.B. Bakker-Van der Jagt:** Writing – review & editing, Investigation, Conceptualization. **A.H. Boer:** Writing – review & editing, Investigation, Conceptualization. **H.M. Ceha:** Writing – review & editing, Investigation, Conceptualization. **J.S. Cnossen:** Writing – review & editing, Investigation, Conceptualization. **M.P.W. Intven:** Writing – review & editing, Investigation, Conceptualization. **M.M. Leseman-Hoogenboom:** Writing – review & editing, Investigation, Conceptualization. **G. Mulder-Ebrahimi:** Writing – review & editing, Investigation, Conceptualization. **K. Muller:** Writing – review & editing, Investigation, Conceptualization. **V. Oppedijk:** Writing – review & editing, Investigation, Conceptualization. **O. Reerink:** Writing – review & editing, Investigation, Conceptualization. **H. Rütten:** Writing – review & editing, Investigation, Conceptualization. **P.H. Spruit:** Writing – review & editing, Investigation, Conceptualization. **B. van Triest:** Writing – review & editing, Writing – original draft, Supervision, Investigation, Data curation, Conceptualization.

## Declaration of competing interest

The authors declare that they have no known competing financial interests or personal relationships that could have appeared to influence the work reported in this paper.

## References

[1] Blay J.Y., Casali P., Ray-Coquard I., Seckl M.J. (2024 Feb). Management of patients with rare adult solid cancers: objectives and evaluation of European reference networks (ERN) EURACAN. Lancet Reg Health Eur.

[2] www.iknl.nl/nkr-cijfers.

[3] Welton ML, Lambert R, Bosman FT. Tumours of the anal canal. In: WHO classification of tumours of the digestive system (4th Ed) Bosman FT, Carneiro F, Rhuban R, Theise N Eds. IARC, Lyon, 2010;184-193.

[4] Glynne-Jones R., Nilsson P.J., Aschele C., Goh V., Peiffert D., Cervantes A. (2014 Jun). Anal cancer: ESMO-ESSO-ESTRO clinical practice guidelines for diagnosis, treatment and follow-up. Radiother Oncol.

[5] D'Souza G., Wiley D.J., Li X., Chmiel J.S., Margolick J.B., Cranston R.D. (2008 Aug 1). Incidence and epidemiology of anal cancer in the multicenter AIDS cohort study. J Acquir Immune Defic Syndr.

[6] Frisch M., Biggar R.J., Goedert J.J. (2000 Sep 20). Human papillomavirus-associated cancers in patients with human immunodeficiency virus infection and acquired immunodeficiency syndrome. J Natl Cancer Inst.

[7] Uronis H.E., Bendell J. (2007). Anal cancer: an overview. Oncologist.

[8] Jimenez W., Paszat L., Kupets R. (2009). Presumed previous human papillomavirus (HPV) related gynecological cancer in women diagnosed with anal cancer in the province of Ontario. Gynecol Oncol.

[9] SEER*Explorer: An interactive website for SEER cancer statistics [Internet]. Surveillance Research Program, National Cancer Institute.

[10] Dewit L, Cats A, Beets G. Evolving Concepts toward Individualized Treatment of Squamous Cell Carcinoma of the Anus. 25 March 2019. DOI: 10.5772/intechopen. 85545.

[11] Nigro N.D., Vaitkevicius V.K., Considine B. (1974). Combined therapy for cancer of the anal canal: a preliminary report. Dis Colon Rectum.

[12] Meulendijks D., Dewit L., Tomasoa N.B., van Tinteren H., Beijnen J.H., Schellens J.H. (2014 Oct 28). Chemoradiotherapy with capecitabine for locally advanced anal carcinoma: an alternative treatment option. Br J Cancer.

[13] Mok H., Briere T.M., Martel M.K. (2011). Comparative analysis of volumetric modulated arc therapy versus intensity modulated radiation therapy for radiotherapy of anal carcinoma. Pract. Radiat Oncol.

[14] Bazan J.G., Hara W., Hsu A. (2011 Aug 1). Intensity-modulated radiation therapy versus conventional radiation therapy for squamous cell carcinoma of the anal canal. Cancer.

[15] Martin D., Balermpas P., Gollrad J., Weiß V.C., Stuschke M., Schäfer H. (2020). RADIANCE-Radiochemotherapy with or without Durvalumab in the treatment of anal squamous cell carcinoma: a randomized multicenter phase II trial. Clin Transl Radiat Oncol.

[16] NCT04046133 Phase 1b/II Trial of Pembrolizumab Plus IMRT in Stage III/IV Carcinoma of Anus (CORINTH) [(accessed on 13 April 2023)]; Available online: https://clinicaltrials.gov/ct2/show/NCT04046133.

[17] NCT03233711 Nivolumab after Combined Modality Therapy in Treating Patients with High Risk Stage II-IIIB Anal Cancer. [(accessed on 16 March 2023)]; Available online: https://clinicaltrials.gov/ct2/show/NCT03233711.

[18] Landelijke werkgroep Gastro-intestinale tumoren. Anuscarcinoom Landelijke Richtlijn, versie 2.0, datum goedkeuring 13-11-2012.

[19] Meulendijks D., Tomasoa N.B., Dewit L., Smits P.H., Bakker R., van Velthuysen M.L. (2015 Apr 14). HPV-negative squamous cell carcinoma of the anal canal is unresponsive to standard treatment and frequently carries disruptive mutations in TP53. Br J Cancer.

[20] Neelis K.J., Kip D.M., Speetjens F.M., van der Linden Y.M. (2022 Apr 20). Treatment results for patients with squamous-cell carcinoma of the anus, a single institution retrospective analysis. Radiat Oncol.

[21] James R.D., Glynne-Jones R., Meadows H.M., Cunningham D., Myint A.S., Saunders M.P. (2013). Mitomycin or cisplatin chemoradiation with or without maintenance chemotherapy for treatment of squamous-cell carcinoma of the anus (ACT II): a randomised, phase 3, open-label, 2 x 2 factorial trial. Lancet Oncol.

[22] Gunderson L.L., Winter K.A., Ajani J.A., Pedersen J.E., Moughan J., Benson A.B. (2012). Long-term update of US GI intergroup RTOG 98–11 phase III trial for anal carcinoma: survival, relapse, and colostomy failure with concurrent chemoradiation involving fuorouracil/mitomycin versus fuorouracil/cisplatin. J Clin Oncol.

[23] Bartelink H., Roelofsen F., Eschwege F., Rougier P., Bosset J.F., Gonzalez D.G. (1997). Concomitant radiotherapy and chemotherapy is superior to radiotherapy alone in the treatment of locally advanced anal cancer: results of a phase III randomized trial of the European Organization for Research and Treatment of Cancer Radiotherapy and Gastrointestinal Cooperative groups. J Clin Oncol.

[24] Ho V.K.Y., Deijen C.L., Hemmes B., van Erning F.N., Snaebjornsson P., van Triest B. (2024 May 1). Trends in epidemiology and primary treatment of anal squamous cell carcinoma in the Netherlands (1990-2021). Int J Cancer.

[25] Frood R., Gilbert A., Gilbert D., Abbott N.L., Richman S.D., Goh V. (2025 Nov 9). Personalising anal cancer radiotherapy dose (PLATO): protocol for a multicentre integrated platform trial. BMJ Open.

[26] Sebag-Montefiore D, Harrison M, Gilbert A, et al. PLATO ACT 4: Long term results of an RCT evaluating reduced dose and standard dose chemoradiotherapy in early-stage anal cancer. ESTRO abstract and presentation 2025.

[27] Lissoni P., Meregalli S., Bonetto E., Mancuso M., Brivio F., Colciago M. (2005 Jul-Dec;19(3–4):153–8.). Radiotherapy-induced lymphocytopenia: changes in total lymphocyte count and in lymphocyte subpopulations under pelvic irradiation in gynecologic neoplasms. J Biol Regul Homeost Agents.

[28] Hyung Joo Baik, Min Sung An, Ji Sun Park, Yunseon Choi. Poor Prognostic Effects of Lymphocytopenia Induced by Preoperative Chemoradiotherapy in Rectal Cancer. Asian Pac J Cancer Prev. 2024 Nov 1;25(11):3799-3805. 10.31557/APJCP.2024.25.11.3799 PMC11996090 39611902

[29] Damen P.J., Kroese T.E., Van Hillegersberg R. (2021). The influence of severe radiation-induced lymphopenia on overall survival in solid tumors: a systematic review and meta-analysis. Int J Radiat Oncol Biol Phys.

