# Supplementary material for: Outcomes after (chemo)radiotherapy for anal cancer – A nationwide cohort study
Source: Clin Transl Radiat Oncol. 2026 Apr 6;59:101163. doi: 10.1016/j.ctro.2026.101163 (PMC13091936; doi:10.1016/j.ctro.2026.101163)
Supplement: Supplementary Data 1 [file mmc1.docx]

**Supplementary materials - Tables**

*Table S1 – Prognostic risk factors of LRFS, univariate versus multi-variable Cox proportional hazards regression*

| Risk Factor | n | Univariate HR  (95% c.i.) | Univariate  p-value | Multi-variable HR (95% c.i.) | Multi-variable  p-value |
| --- | --- | --- | --- | --- | --- |
| Age (per 10 years) | 457 | 1.09 (0.91-1.29) | n.s. |  |  |
| Male sex | 457 | 1.93 (1.27-2.92) | ** | 1.00 (0.41-2.42) | n.s. |
| Caseload per annum  < 25 cases  ≥ 25 cases  ≥ 40 cases | 454 | (baseline)  1.43 (0.78-2.62)  0.97 (0.64-1.48) | n.s.  n.s. |  |  |
| WHO score ≥ 2 | 449 | 1.62 (0.84-3.13) | n.s. |  |  |
| T stage ≥ 3 | 457 | 1.52 (1.00-2.29) | * | 0.93 (0.29-2.99) | n.s. |
| N stage ≥ 1 | 453 | 1.29 (0.86-1.95) | n.s. |  |  |
| Overall stage  I and II  ≥ IIIA  ≥ IIIB | 453 | (baseline)  1.22 (0.80-1.84)  1.71 (1.13-2.60) | n.s.  * | 1.82 (0.72-4.60) | n.s. |
| No chemotherapy | 457 | 2.18 (1.35-3.52) | ** | 1.46 (0.30-7.14) | n.s. |
| Max tumour length | **453** | **1.25 (1.14-1.37)** | ******* | **1.43 (1.10-1.87)** | ****** |
| Primary tumour dose per 10Gy EQD2 | 457 | 1.14 (0.61-2.13) | n.s. |  |  |
| Elective nodes dose per 10Gy EQD2 | 421 | 1.70 (1.00-2.69) | * | 1.39 (0.52-3.74) | n.s. |
| HIV positive | 185 | 1.28 (0.53-3.09) | n.s. |  |  |
| HPV negative | **155** | **2.92 (1.38-6.21)** | ****** | **4.22 (1.63-10.9)** | ****** |
| SCC level ≥ 1.5 | 38 | 6.15 (0.74-51) | n.s. |  |  |

*Abbreviations and symbols used in the table : 95% c.i.:95% confidence interval; n.s.: not statistically significant; *: p<0.05; **: p<0.01; ***: p<0.001; WHO: World Health Organization; EQD2: equivalent radiotherapy dose in units of 2Gy per fraction; HIV: human immunodeficiency virus; HPV: human papilloma virus; SCC: squamous cell carcinoma; HR: hazard ratio.*

*Table S2 – Prognostic risk factors of OS, univariate versus multi-variable Cox proportional hazards regression*

| Risk Factor | n | Univariate HR  (95% c.i.) | Univariate  p-value | Multi-variable HR (95% c.i.) | Multi-variable  p-value |
| --- | --- | --- | --- | --- | --- |
| Age (per 10 years) | 457 | 1.34 (1.16-1.55) | *** | 1.11 (0.82-1.48) | n.s. |
| Male sex | 457 | 1.67 (1.21-2.32) | ** | 1.31 (0.68-2.53) | n.s. |
| Caseload per annum  < 25 cases  ≥ 25 cases  ≥ 40 cases | 454 | (baseline)  0.91 (0.60-1.39)  0.77 (0.55-1.08) | n.s.  n.s. |  |  |
| WHO score ≥ 2 | **449** | **3.04 (1.96-4.73)** | ******* | **3.32 (1.1-10)** | ***** |
| T stage ≥ 3 | 457 | 1.52 (1.09-2.11) | * | 0.91 (0.36-2.29) | n.s. |
| N stage ≥ 1 | 453 | 1.34 (0.97-1.86) | * | 0.56 (0.14-2.16) | n.s. |
| Overall stage  I and II  ≥ IIIA  ≥ IIIB | 453 | (baseline)  1.30 (0.94-1.80)  **1.91 (1.38-2.66)** | n.s.  ******* | **3.49 (0.95-12.8)** | ***** |
| No chemotherapy | 457 | 1.66 (1.11-2.48) | * | 2.66 (0.94-7.51) | n.s. |
| Max tumour length | 453 | 1.19 (1.11-1.29) | *** | 1.20 (0.98-1.46) | n.s. |
| Primary tumour dose per 10Gy EQD2 | 457 | 1.01 (0.65-1.57) | n.s. |  |  |
| Elective nodes dose per 10Gy EQD2 | 421 | 1.51 (1.05-2.18) | * | 1.02 (0.48-2.18) | n.s. |
| HIV positive | 185 | 0.64 (0.27-1.48) | n.s. |  |  |
| HPV negative | **155** | **2.97 (1.66-5.29)** | ******* | **3.09 (1.44-6.65)** | ****** |
| SCC level ≥ 1.5 | 38 | 7.05 (0.87, 57) | n.s. |  |  |

*Abbreviations and symbols used in the table : 95% c.i.:95% confidence interval; n.s.: not statistically significant; *: p<0.05; **: p<0.01; ***: p<0.001; WHO: World Health Organization; EQD2: equivalent radiotherapy dose in units of 2Gy per fraction; HIV: human immunodeficiency virus; HPV: human papilloma virus; SCC: squamous cell carcinoma; HR: hazard ratio.*

*Table S3 – Prognostic risk factors of CFS, univariate versus multi-variable Cox proportional hazards regression*

| Risk Factor | n | Univariate HR  (95% c.i.) | Univariate  p-value | Multi-variable HR (95% c.i.) | Multi-variable  p-value |
| --- | --- | --- | --- | --- | --- |
| Age (per 10 years) | 407 | 1.08 (0.86-1.36) | n.s. |  |  |
| Male sex | 407 | 1.63 (0.96-2.79) | n.s. |  |  |
| Caseload per annum  < 25 cases  ≥ 25 cases  ≥ 40 cases | 404 | (baseline)  3.23 (1.01-10.3)  1.42 (0.84-2.41) | *  n.s. | 2.70 (0.33-22.3) | n.s. |
| WHO score ≥ 2 | **399** | **3.26 (1.52-6.97)** | ****** | **6.60 (1.60-27.3)** | ****** |
| T stage ≥ 3 | 407 | 2.51 (1.48-4.26) | *** | 1.22 (0.32-4.64) | n.s. |
| N stage ≥ 1 | 403 | 1.26 (0.74-2.15) | n.s. |  |  |
| Overall stage  ≥ IIIA  ≥ IIIB | 403 | 1.30 (0.76-2.22)  1.84 (1.07-3.17) | n.s.  ** | 2.11 (0.76-5.86) | n.s. |
| No chemotherapy | 407 | 1.21 (0.57-2.55) | n.s. |  |  |
| Max tumour length | **404** | **1.35 (1.20-1.52)** | ******* | **1.37 (1.02-1.84)** | ***** |
| Primary tumour dose per 10Gy EQD2 | 407 | 2.31 (0.96-5.55) | n.s. |  |  |
| Elective nodes dose per 10Gy EQD2 | 375 | 1.70 (0.96-3.02) | n.s. |  |  |
| HIV positive | 168 | 1.26 (0.48-3.30) | n.s. |  |  |
| HPV negative | **145** | **5.69 (2.38-13.6)** | ******* | **11.7 (4.07-33.7)** | ******* |
| SCC level ≥ 1.5 | 35 | 4.16 (0.46-37.3) | n.s. |  |  |

*Abbreviations and symbols used in the table : 95% c.i.:95% confidence interval; n.s.: not statistically significant; *: p<0.05; **: p<0.01; ***: p<0.001; WHO: World Health Organization; EQD2: equivalent radiotherapy dose in units of 2Gy per fraction; HIV: human immunodeficiency virus; HPV: human papilloma virus; SCC: squamous cell carcinoma; HR: hazard ratio.*

*Table S4 - Prognostic risk factors of CR within 6 months, univariate versus multi-variable logistic regression*

| Risk Factor | n | Univariate OR  (95% c.i.) | Univariate  p-value | Multi-variable OR (95% c.i.) | Multi-variable  p-value |
| --- | --- | --- | --- | --- | --- |
| Age (per 10 years) | 422 | 1.11 (0.92-1.34) | n.s. |  |  |
| Male sex | 422 | 0.63 (0.40-1.00) | * | 0.79 (0.30-2.09) | n.s. |
| Caseload per annum  < 25 cases  ≥ 25 cases  ≥ 40 cases | 419 | (baseline)  0.79 (0.40-1.45)  0.57 (0.36-0.90) | n.s.  * | 0.48 (0.17-1.29) | n.s. |
| WHO score ≥ 2 | 414 | 0.48 (0.23-1.04) | n.s. |  |  |
| T stage ≥ 3 | 422 | 0.49 (0.31-0.79) | ** | 0.61 (0.16-2.31) | n.s. |
| N stage ≥ 1 | 418 | 0.75 (0.47-1.19) | n.s. |  |  |
| Overall stage  ≥ IIIA  ≥ IIIB | 418 | 0.68 (0.43-1.08)  0.55 (0.34-0.89) | n.s.  * | 0.74 (0.27-2.09) | n.s. |
| No chemotherapy | 422 | 1.03 (0.55-2.02) | n.s. |  |  |
| Max tumour length | 419 | 0.85 (0.76-0.95) | ** | 0.82 (0.59-1.11) | n.s. |
| Primary tumour dose per 10Gy EQD2 | 422 | 1.22 (0.63-2.24) | n.s. |  |  |
| Elective nodes dose per 10Gy EQD2 | 387 | 0.44 (0.23-0.77) | ** | 0.39 (0.09-1.32) | n.s. |
| HIV positive | 179 | 0.60 (0.24-1.56) | n.s. |  |  |
| HPV negative | 144 | 0.38 (0.16-0.92) | * | 0.33 (0.10-1.07) | n.s. |
| SCC level ≥ 1.5 | 37 | 0.35 (0.08-1.32) | n.s. |  |  |

*Abbreviations and symbols used in the table : 95% c.i.:95% confidence interval; n.s.: not statistically significant; *: p<0.05; **: p<0.01; ***: p<0.001; WHO: World Health Organization; EQD2: equivalent radiotherapy dose in units of 2Gy per fraction; HIV: human immunodeficiency virus; HPV: human papilloma virus; SCC: squamous cell carcinoma; OR: odds ratio.*

*Table S5. Toxicity – acute*

|  |  |
| --- | --- |
|  | **N** |
| Dermatitis peri-anal  Grade 3  Grade 4 | 241  1 |
| Dermatitis inguinal  Grade 3  Grade 4 | 115  0 |
| Diarrhea  Grade 3  Grade 4 | 33  0 |
| Proctitis  Grade 3  Grade 4 | 29  1 |
| Fecal incontinence  Grade 3  Grade 4 | 7  0 |
| Cystitis  Grade 3  Grade 4 | 1  0 |
| Treatment for short term toxicity  Wound care  Pain medication  Medication (other than pain medication)  Stoma  Other | 317  304  55  2  21 |

*Table S6. Toxicity – late*

|  |  |
| --- | --- |
|  | **N** |
| Dermatitis peri-anal  Grade 3  Grade 4 | 6  1 |
| Dermatitis inguinal  Grade 3  Grade 4 | 1  0 |
| Diarrhea  Grade 3  Grade 4 | 3  1 |
| Proctitis  Grade 3  Grade 4  Grade 5 | 38  1  1 |
| Fecal incontinence  Grade 3  Grade 4 | 17  0 |
| Cystitis  Grade 3  Grade 4 | 2  0 |
| Oedema limbs  Grade 3  Oedema laterality:  *Unilateral*  *Bilateral* | 6  *2*  *4* |
| Insufficiency fractures  Reported  Fracture location:  *Os sacrum*  *Os ilium*  *Os ischium*  *Os pubis*  *Other location / complex fractures* | 17  *12*  *0*  *0*  *1*  *4* |
| Pain – requiring medication  Reported  Pain location:  *Abdominal only*  *Local only*  *Local and abdominal*  *Other location* | 39  *4*  *22*  *3*  *10* |
| Anal stenosis  Grade 3  Grade 4 | 10  1 |
| Anal ulcer  Grade 3  Grade 4 | 14  0 |
| Female dyspareunia  Yes  No  Not reported | 30  157  57 |
| Erectile dysfunction  Yes  No  Not reported | 17  153  42 |
| Urinary incontinence  Grade 3  Grade 4 | 1  0 |
| Treatment for long term toxicity  Pain medication  Medication (other than pain medication)  Hyperbaric O2  Stoma  Wound care  Support stockings  Lymph drainage  Other | 45  26  20  19  16  5  2  50 |
